# Predictors for HIV testing among Chinese workers in infrastructure construction enterprises in Kenya

**DOI:** 10.1186/s12889-021-12234-1

**Published:** 2021-12-04

**Authors:** Wenjuan Zhou, Wenyu Deng, Junfei Luo, Yin Bai, Zeyi He, Honghong Wang

**Affiliations:** 1 School of literature and journalism, Central South University, Changsha, Hunan Province China; 2 Xiang Ya Nursing School, Central South University, Changsha, Hunan Province China

**Keywords:** HIV testing, Sexual attitude, Transnational migrants, Construction workers

## Abstract

**Background:**

There are increasing Chinese migrants in sub-Saharan Africa currently. Most of them are engaged in infrastructure construction. Research has shown that they stay at particular risk of HIV and are recommended for HIV testing. However, their HIV testing behavior, and its relevant factors, have not been researched among them by now. This study describes the recent HIV testing behavior and relevant factors among Chinese migrant workers in Kenya.

**Methods:**

A cross-sectional survey was conducted among 110 male Chinese workers from six different Chinese infrastructure construction enterprises in Kenya. Furthermore, a two-stage cluster random sampling method was used to select participants. We used a questionnaire that included HIV testing history, demographic characteristics, and putative multilevel facilitators of HIV testing. Logistic regression was used to explore the predictors of recent HIV testing behavior among Chinese migrant workers in Kenya.

**Result:**

Of the 110 participants, 30 (27.27%) were tested for HIV in the recent year. All participants were male, and the majority were married (73.2%). The mean age was 37.49 years (SD = 9.73; range: 23 to 63), and a considerable proportion refused to answer questions about transactional sexual behaviors in the last year. Most were able to obtain HIV-related information (91.8%) and were exposed to HIV-related information in the last year (68.2%), but only 47.6% had sufficient HIV knowledge. Nearly one-fifth of them believed that selling sex and paying for sex is acceptable. Multiple logistic regression analysis indicated that participants who could accept the ‘pay for sex’ (OR: 2.74; 95% CI: 1.02, 7.36) and exposed to HIV related information (OR: 4.75; 95% CI: 1.29, 17.44) were more likely to test for HIV in the recent 1 year.

**Conclusion:**

Higher current HIV test rates were associated with a more open sexual attitude towards paying for sex and being exposed to HIV-related information in the last year among Chinese workers in Kenya. More specific attention to HIV should be attached to this population to increase the rate of HIV testing among them.

**Supplementary Information:**

The online version contains supplementary material available at 10.1186/s12889-021-12234-1.

## Background

According to United Nations AIDS agency (UNAIDS), 37.9 million people were living with the human immunodeficiency virus (HIV) worldwide by 2019, and more than two-thirds of these cases are in sub-Saharan Africa [1]. Kenya, located in eastern Africa, is one of the most politically stable and economically sound countries in sub-Saharan Africa and is seriously affected by the HIV epidemic [2]. Since China and Kenya formally established diplomatic relations in 2013, and with the Belt and Road initiative in China [3], there are increasing Chinese migrants in Africa and Kenya. By the end of 2019, more than 200,000 Chinese migrants in Africa and nearly 10,000 Chinese migrants in Kenya, the majority of whom were workers on infrastructure construction [4].

This expanding group is encountering a kind of disproportionate HIV risk. A meta-analysis by Armstrong Dzomba et al. in Africa demonstrated that the risk of HIV infection for immigrants is 1.69 times that of non-immigrants [5]. One study using European Surveillance System data in 2007-2012 found that 38% of HIV cases in the European Union/European Economic Area were migrant workers and over 50% of these migrants from Africa [6]. In China, migrant workers are also considered to be a high-risk population for HIV infection and play a vital role in fuelling the HIV epidemic [7, 8]. According to a survey of epidemics in China, migrant workers coming back from Africa are more likely to have HIV than the general population [9]. Previous studies have demonstrated multilevel determinants of HIV infection among them, which included socio-economic vulnerability as age, sex, and nationality [10–12]; migrant work and lifestyle factors such as staying away from stable sexual partners [13], policy, and structural context as the limited access to health care [10].

In September 2019, the State Council of China promulgated an implementation plan to curb the spread of AIDS (2019-2022), which mentioned the extreme necessity to improve the willingness of a high-risk population, including migrants workers in Africa, to proactively engage with a high-risk population test for HIV [8]. Chinese migrant workers in Africa can voluntarily get HIV testing for free [8]. There is no doubt that HIV testing possesses significant public health implications in HIV risk control and other health services [14, 15]. It is the entry point for HIV treatment, care, and support services [16, 17]. Plenty of factors can affect HIV testing behaviors in critical populations. Previous studies among them have found some factors including demographic factors as age, marriage, educational level [18]; structural factors as routine medical check-ups, accessibility of HIV testing, affordable health care [19, 20]; a previous experience like HIV related educational attainment, HIV related cognition, previous HIV testing experience [21, 22]; HIV risk perception and other factors as sexual attitudes, risky sexual behaviors [12, 23]. Studies among migrant workers in high-income countries found that they might pose additional difficulties in HIV testing, such as language, administrative, legal, and cultural barriers [12]. As the profound influence of Confucianism, the Chinese are considerably conservative to sex topics. Chinese unique sexual attitude may also be one part of factors influencing HIV testing [24]. Unfortunately, no study about HIV testing has targeted Chinese migrant workers in Africa where shoulders the heaviest burden of HIV worldwide. This study aims to describe recent HIV testing behavior and associated factors in this group and then identify effective intervention strategies for HIV testing.

## Methods

### Design, settings, and sample

This cross-sectional study was conducted among male Chinese workers (rare female workers) in Kenya from six different Chinese infrastructure construction companies. A two-stage cluster sampling method was used to select participants. In the first stage, we randomly selected six companies from the online list of 63 Chinese infrastructure construction enterprises in Kenya. All the six construction companies have contracted for major infrastructure projects in Kenya, such as railways, roads, and bridges. In the second stage, we obtained information about projects of each selected enterprise by inquiring about the enterprise’s website and then randomly selected 1-2 projects out of them. The anonymous investigation was completed by all qualified Chinese employees of the selected projects. A participant must have Chinese nationality and have work experience in Kenya for at least 6 months to be eligible. We used G*power software to calculate the sample size. The results showed that the minimum sample size was 104 cases (the effect size was 0.31, the significance level α was 0.05, and the test power 1-β was 0.9) [25, 26]. As all the participants were Chinese, the questionnaire was available in Mandarin. All participants at least have a primary school educational background, and the investigator was on site to ensure that all participants fully understood the questionnaire. After the researcher explained the purpose of the study to the participants and obtained informed consent from them, participants filled in the questionnaire on paper. Fifteen minutes later, the questionnaires were collected by a trained investigator on site. In the end, 124 Chinese workers who worked for six different Chinese infrastructure construction enterprises in three different regions of Kenya (Nairobi, Machakos, and Mombasa) were eligible, and 110 of them submitted questionnaires.

### Measures

#### Demographic characteristics and HIV testing behavior

Demographic characteristics regarding the workers’ age, marriage, education level, residence, work position (the position determines the status within the organization and the income level, but all of them earn more than $ 20,000 per year), the experience of working away from home in China and of working abroad was collected. The key outcome variable of this study was whether they had any form of HIV testing in the past year. The following questions were asked: ‘Did you take an HIV test last year?’. The response options were ‘Yes’ or ‘No’.

#### HIV knowledge

We used the knowledge section of the Chinese National AIDS Sentinel Surveillance Questionnaire explicitly designed for overseas migrant workers to assess their level of knowledge on HIV [8]. There are 13 items in this questionnaire, in which one score is set for each correct answer, and a total score of 10 or higher indicates sufficient HIV knowledge. The internal reliability (KR-20) of this HIV knowledge questionnaire is 0.64, which suggests good internal consistency.

#### HIV risk perception and related factors

According to previous literature [24], participants were asked, “Do you think you might be infected with HIV?’ with responses ranging from 1 (Possible) to 3 (Not Possible), to assess the perceived risk to HIV. We also collected data on HIV risk perception-related factors: sexual attitudes and sexual behaviors. Sexual attitudes were assessed with the brief sexual attitudes scale [27]. The sexual attitudes questionnaire included attitudes toward extramarital sex, selling sex, paid sex, multiple sexual partners, and premarital sex. Participants rated items ranging from 1 (completely unacceptable) to 5 (completely acceptable). For each item, scores lower than 4 were considered ‘unacceptable’, and scores of 4 and higher were considered ‘acceptable’. The Cronbach’s alpha for the scale was 0.84. Sexual behaviors were assessed with the Chinese National AIDS Sentinel Surveillance Questionnaire about sexual behavior explicitly designed for overseas migrant workers [8]. The workers were asked about their commercial sexual behaviors, the number of sexual partners in the last year and condom used behaviors. They have the option to record ‘deadline to answer.

#### Cues to action

We designed one question to evaluate cues to action based on literature [24] ‘Have you received any form of HIV-related information last year?’. The response options were ‘Yes’ or ‘No’. If the participant answered yes, he was considered exposed to HIV-related information in the past year.

#### Accessibility of HIV testing

We designed two questions to evaluate the accessibility of HIV testing for workers based on literature [24, 28]:(1) Do you think you are capable of obtaining relevant information? (2) Do you know where to go for HIV testing? Response options were ‘Yes ‘or’ No’.

#### Analyses

The software SPSS23.0 was used to analyze the data. The measurement data were expressed as mean ± standard deviation and the counting data as cases and percentages. To analyze the association between HIV testing behavior and various factors in the past year, the Logistic regression analysis was used. A backward conditional method was used to build the multivariate logistic regression model. All statistical analyses were double-tailed, with a mean level of 0.05 indicating statistical significance.

## Result

### Demographic characteristics and HIV testing behavior

The demographic characteristics of the participants are shown in Table 1. These participants were from 23 to 63 years old (mean = 37.49 years; SD = 9.725), and most of them were married (73.2%). About 48.2% had primary education, 17.3% had a secondary education, and 34.5% had a higher education level. As for their work position, 34.9% were administrative staff, 27.4% were technical personnel, 26.4% were ordinary workers, and 11.3% were logistical personnel. 45.5% were from urban districts, 34.5% were from rural districts, and 20% suburban districts. As for migration experiences, 32.7% of them had ever worked away from home in China for more than 5 years, and 21.8% had ever worked abroad for more than 5 years. About 27% of our participants received HIV testing in the last year.

**Table 1:** Demographic characteristics

Item	Category	Number	Percent
Age Group			
	<30	35	33
	31-45	46	43.4
	>45	25	23.6
Marital Status			
	unmarried	26	24.1
	married	79	73.2
	cohabitation	2	1.9
	divorced	1	0.9
Educational Background			
	primary education	53	48.2
	secondary education	19	17.3
	higher education	38	34.5
Work Position			
	administrative staff	37	34.9
	technical personnel	29	27.4
	ordinary workers	28	26.4
	logistical personnel	12	11.3
Residency			
	urban	50	45.5
	sub-urban	22	20
	rural	38	34.5
Experience of working away from home in China			
	< 5 years	74	67.3
	≥5 years	36	32.7
Experience of working abroad			
	< 5 years	86	78.2
	≥5 years	24	21.8
Received HIV test in recent one year			
	Yes	30	27.3
	No	80	72.7

### HIV knowledge

Only 48.1% of the participants had sufficient HIV knowledge. Two questions with the lowest correct rates were ‘HIV was equal to death’ and ‘whether HIV antibody could be detected within one week after infection’ (respectively 24.3 and 34.0%). The following two questions with correct low rates were “whether one could be infected by kissing HIV-positive people” and “whether you can tell if someone has HIV by looking at them” (respectively 52.4 and 56.1%).

### HIV risk perception and related factors

Table 2 reports descriptive statistics on HIV-related perceptions and related factors. Mere 1.8% of the participants thought that they were possible to infect HIV. As for sexual attitudes, participants considered it unacceptable to have multiple sexual partners or have an affair with sexual partners (respectively 83.5 and 89.0%). However, they held relatively tolerant attitudes towards paying for sex and selling sex, which was reported acceptable by nearly 20% of the participants. Even the concept of accepting one-night stand behavior won 25.0% among participants. Most participants (71.6%) showed an acceptable attitude towards premarital sex. As for questions, most participants refused to answer the question about transactional sex in Kenya, and the rest, only 5.4% of them, reported that they had transactional sex in Kenya. More than half of the participants reported only one sexual partner in the last year, half of them used condoms at last intercourse, 22.7% of them always used condoms in the last 12 months.

**Table 2:** Characteristics of the sample

Item	Validated Question	Category	Number	Percent
HIV knowledge	Yes^a^			
		Sufficient	53	48.2
		Insufficient	57	51.8
Perceived risk of HIV	No^b^			
		Possible	2	1.9
		Little possible	23	21.5
		Not possible	82	76.6
Sexual attitude	Yes^c^			
premarital sex		Acceptable	78	71.6
		Not	31	28.4
Extramarital sex		Acceptable	18	16.5
		Not	91	83.5
Paying for sex		Acceptable	24	21.8
		Not	86	78.2
Selling sex		Acceptable	20	18.5
		Not	88	81.5
Multiple sexual partners		Acceptable	12	11.9
		Not	97	88.1
One-night stand		Acceptable	27	24.5
		Not	81	73.6
Sexual behaviors	Yes^a^			
Paying money for sex		Yes	6	5.4
		No	12	11
		Refuse to answer	92	83.6
No. of sexual partners in last year		0	23	20.9
		1	60	54.5
		>2	9	16.2
		Refuse to answer	18	32.4
Condom use at last intercourse		Yes	55	50
		No	39	36.5
		Refuse to answer	16	14.5
Frequency of condom use in the last year		never	20	18.2
		sometimes	19	17.3
		often	23	20.9
		always	25	22.7
		Refuse to answer	23	20.9
Received HIV Related Information in recent one year	No^b^			
		Yes	75	68.2
		No	35	31.8
The capability of obtaining HIV-related information	No^b^			
		Yes	101	91.8
		No	9	8.2
Information about sites for receiving HIV test	No^b^			
		Yes	105	95.5
		No	5	4.6

^a^The item was adapted from the Chinese Unified National AIDS Sentinel Surveillance Questionnaire explicitly designed for overseas migrant workers

^b^ The item was developed for the current study

^c^The item was adapted from the brief sexual attitudes scale

### Cues to action

In terms of cues to action, more than half of the participants (68.2%) received HIV-related information in the last year.

### Accessibility of HIV testing services

Most of the participants could obtain HIV-related information (91.8%) and information about sites for receiving HIV testing (95.5%).

### Factors associated with recent HIV testing

We used multivariate logistic regression and identified two variables associated with recent HIV testing. They were exposure to HIV related information in recent 1 year (OR: 4.75; 95% CI: 1.29, 17.44; *P* = 0.019) and acceptable attitudes towards paying for sex (OR: 2.74; 95% CI: 1.02, 7.36; *P* = 0.046).

## Discussion

To our knowledge, it is the first research that tried to describe HIV testing behavior and identify the possible associated factors among Chinese migrant construction workers in Kenya. Few studies about HIV testing have focused on this group, although they are continuously expanding and are more likely to come down with HIV than the general population. This study demonstrated that 27% of them had been tested for HIV last year. It is lower than 36 to 47% found in population-level in Kenya [29] and 73% in local construction workers in South Africa by Bowen et al [30] Thus it is essential to know the factors associated with HIV testing behavior among them and comprehend the reasons averting them from taking the test.

The great majority of participants were under 45 years old(76.4%), which was a sexually active segment and usually had to work continuously for more than 1 year in Africa. Previous research has revealed that sexually active young adults who are separated from their stable sexual partners for a long time may increase the likelihood of HIV/sexually transmitted diseases [31, 32]. With the scale-up HIV prevention services among critical populations all over the world, most participants were accessible to obtain HIV-related information (91.8%) and messages about sites for receiving HIV tests (95.5%) in our study, which were reported to be facilitators to uptake of HIV testing in many studies [22]. However, more than half of our sample reported a lack of HIV knowledge and cognition. The gap between their capability to obtain HIV-related information and HIV-related cognition might be related to low education levels. Most participants were from rural areas and only received primary education (e.g., elementary, middle school), limiting their ability to understand HIV-related information [33].

Among a set of sex attitudes, the open attitude toward paying for sex was the most influential with recent HIV testing behavior. It has been shown among internal migrant construction workers in China that people who are more open to paying for sex are more likely to have transactional sex [34]. Although we collected information about workers’ sexual behavior in the past year, up to 83.6% of them refused to answer the question about sexual partners, including transactional sex. Their resistance to this question could be attributed to their sensitive identity as workers of overseas Chinese companies and conservative culture to sex. The previous studies have demonstrated a clear link between risky sexual behaviors (e.g., having paid sex) and recent HIV testing [35–40]. It can be inferred that participants who have more open attitudes of paid sex are more likely to have risky sexual behavior, which can raise the perception of HIV risk and contribute to an increased likelihood of HIV testing. Therefore, we need more exploration among this population to obtain more information about the risks of sexually transmitted diseases.

What is more, we observed an association between recent exposure to HIV-related information and HIV testing behaviors. This positive association has been described in previous studies among youth in Sub-Saharan Africa [41]. According to Health Belief Model (HBM), cues to action refer to the enabling factors for people to take preventive measures, including media campaigns, medical staff reminders, others’ advice, family, and friends’ disease information, etc [42] All the above that expose the workers to HIV-related information will remind them of the risk of HIV and the need to act as a test for HIV to mitigate the risk [43–45]. This approach suggests a need for continuous HIV-related information targeted toward transnational migrant construction workers at their working sites and leisure sites to improve HIV knowledge and uptake of HIV testing.

There exist a couple of notable strengths in our study. First, we focused on Chinese migrant workers in Africa, a group easier to engage in HIV risky sexual behavior but often ignored. Second, we randomly selected six different companies for sampling, which was able to reduce sample bias. Thus, we believe our data was both reliable and valid. Third, we have directly confirmed the correlations between sexual attitude and HIV testing behavior for the first time.

Nevertheless, our study still also has limitations. 1: the three items of the questionnaires (accessibility of HIV testing services, cues to action, and HIV risk perception) were designed by the researchers based on research purpose and literature review. These questions have been used for studies among Chinese undergraduate students, but we did not carry out a pilot study to further verify its applicability among Chinese workers in Africa. 2: all the data were self-reported and might increase the possibility for social desirability bias, especially the data on risky sex. Future studies with more standardized and valid measurements are expected to provide substantial evidence for developing effective HIV services for Chinese migrant workers in Africa.

## Conclusion

This study reported recent HIV testing behavior and related factors among Chinese migrant construction workers in Kenya who have not been thoroughly studied but faced disproportionate HIV risk currently. We found that the open sexual attitude among them, which maybe predict the possibility of risky sexual behaviors (e.g., having paid sex), will further influence their HIV testing behavior. Various HIV/AIDS information will also inform them of their risk of STIs/HIV and increase their HIV testing rates.

## Supplementary Information

**Additional file 1:.**

## Data Availability

The datasets used and/or analyzed during the current study are available from the corresponding author on reasonable request.

## Abbreviations

HIV: Human immunodeficiency virus
UNAIDS: The Joint United Nations Program on HIV/AIDS
STIs: Sexually transmitted diseases
