# Supplementary material for: Predictors for HIV testing among Chinese workers in infrastructure construction enterprises in Kenya
Source: BMC Public Health. 2021 Dec 4;21:2213. doi: 10.1186/s12889-021-12234-1 (PMC8642875; doi:10.1186/s12889-021-12234-1)
Supplement: Supplementary file 1 — Additional file 1:. [file 12889_2021_12234_MOESM1_ESM.docx]

**1、 Basic information:**

1. Gender: ① male and ② female

2. Age (please fill in): years old

3. Marital status: ① unmarried; ② married; ③ cohabitation; ④ divorce

4. Education level: ① primary school ② secondary school ③ technical secondary school ④diploma education⑤ Undergraduate ⑥ graduated education

5. The location of your family in China: ① urban; ② sub-urban; ③ town; ④ rural

6. Your position: ① administrative staff ② Technical personnel ③ ordinary workers ④ logistics personnel

7. Your working years in the current enterprise: ① less than 5 years; ② 5 to 10 years; ③ 10 to 20 years; ④ more than 20 years

8. Before you came to Kenya, did you have overseas work experience: ① yes: Please fill in the years of service ; ② No

9. When you are in China, do you have any experience of working abroad: ① yes: Please fill in the years ② No

10. Enterprises (please fill in)

**2、 Attitudes towards sex:**

1. Do you think premarital sex happens

① It is totally acceptable; ② the majority is acceptable; ③ it is basically acceptable

④ A few can be accepted; ⑤ totally unacceptable

2. Do you think extramarital sex has happened

① It is totally acceptable; ② the majority is acceptable; ③ it is basically acceptable

④ A few can be accepted; ⑤ totally unacceptable

3. Do you think spending money to have sex with others

① It is totally acceptable; ② the majority is acceptable; ③ it is basically acceptable

④ A few can be accepted; ⑤ totally unacceptable

4. Do you think you get money by having sex with others

① It is totally acceptable; ② the majority is acceptable; ③ it is basically acceptable

④ A few can be accepted; ⑤ totally unacceptable

5. Do you feel that you have sex with more than one person at the same time

① It is totally acceptable; ② the majority is acceptable; ③ it is basically acceptable

④ A few can be accepted; ⑤ totally unacceptable

6. Do you think one night stand

① It is totally acceptable; ② the majority is acceptable; ③ it is basically acceptable

④ A few can be accepted; ⑤ totally unacceptable

**3、 HIV testing behavior**

1. Did you take an HIV test in the last year? ①Yes ②No

2. When was your last test?

① Never detected; ② within one month; ③ within three months; ④ within half a year; ⑤ within one year; ⑥within three years; ⑥ within five years; ⑧ more than five years

3.Do you think you might be infected with AIDS?

①possible ②impossible ③little possibility

4. Do you think you might be infected with other STDs?

①possible ②impossible ③little possibility

**4、 The situation of receiving AIDS publicity and education:**

1. Have you exposed to HIV related information in recent one year？ ① yes, ② No

2. Do you think you are capable of obtaining relevant information？① yes, ② No

3.Do you know where to go for HIV testing？① yes, ② No

**5、 AIDS related knowledge**

1.Is it possible to judge whether a person is infected with AIDS？ ①Yes ②No ③Not sure

2. Will mosquito bites spread AIDS? ①Yes ②No ③Not sure

3. Will eating with HIV infected people or patients be infected with AIDS？ ①Yes ②No ③Not sure

4. Can I get AIDS by inputting blood with HIV？①Yes ②No ③Not sure

5. Is it possible to get AIDS by sharing syringes with HIV infected people？①Yes ②No ③Not sure

6. Is it possible for children born to HIV infected women to get AIDS？①Yes ②No ③Not sure

7. Is it possible to get AIDS by kissing people with HIV (saliva exchange, no oral wound)? ①Yes ②No ③Not sure

8. Is it possible to get AIDS by having unprotected sex (without using condoms) with an HIV-infected person?①Yes ②No ③Not sure

9. Can proper condom use reduce the spread of AIDS？①Yes ②No ③Not sure

10. Can having sex with a fixed partner reduce the spread of AIDS?

①Yes ②No ③Not sure

11. After HIV infection, will the body's natural resistance to disease decline?

①Yes ②No ③Not sure

12.Does AIDS mean death？①Yes ②No ③Not sure

13. Can antibodies be detected in the blood within one week after HIV infection？

①Yes ②No ③Not sure

**6、** **Sexual behaviors**

1．Have you paid money for sex in the past year？

①Yes ②No

2. Number of sex partners in last one year (please fill in)

3. How often do you use condoms in the past year？

① never ② sometimes③ often④ always

4.Did you use a condom during your last sex？

①Yes ②No
